# Yinchenhao decoction for chronic hepatitis B: Protocol for a systematic review and meta-analysis

**DOI:** 10.1097/MD.0000000000014648

**Published:** 2019-02-22

**Authors:** Lu Xu, Tian Xie, Tao Shen, Shengnan Jian

**Affiliations:** a School of Basic Medicine, Chengdu University of Traditional Chinese Medicine, Chengdu, Sichuan; b Shenzhen Hospital, Guangzhou University of Chinese Medicine, Guangzhou, Guangdong, China.

**Keywords:** chronic hepatitis B, protocol, systematic review, Yinchenhao decoction

## Abstract

**Background:**

Chronic hepatitis B (CHB) is a very critical threat to public health worldwide, and is the primary cause of chronic hepatitis, cirrhosis, and hepatocellular carcinoma (HCC). Yinchenhao decoction (YCHD) is a classic prescription of traditional Chinese medicine for the treatment of jaundice. Despite the satisfactory clinical efficacy of YCHD in the treatment of CHB, the safety of YCHD is still uncertain. Therefore, we will provide a systematic review of YCHD in CHB treatments.

**Methods:**

The purpose of this review is to retrieve relevant literature on YCHD therapy for CHB in the electrical databases, including 4 Chinese databases (e.g., Wanfang database, Chinese National Knowledge Infrastructure (CNKI), Chinese Biomedical database (CBM), Chinese Science and Technology Periodical database (VIP)) and 3 English databases (e.g., PubMed, Cochrane Library, and EMBASE). The literatures involved are from the establishment of the databases to January 2019. The primary outcomes are HBV-DNA, HBeAg, and ALT. We will calculate the data synthesis for meta-analysis using RevMan V.5.3 software if the results are suitable.

**Results:**

This study will offer a high-quality composite of existing evidence for YCHD in CHB treatment according to HBV-DNA quantitative detection, HBeAg qualitative detection, ALT levels, TCM syndrome evaluation criteria, status changes in quality of life assessment participants, and adverse events.

**Conclusion:**

This systematic review will provide evidence to assess the clinical efficacy of YCHD in the treatment of patients with CHB.

**PROSPERO registration number:**

PROSPERO CRD42019119720.

## Abstract 2

Supplemental Digital Content is available in the text

## 1 Introduction

Chronic hepatitis B (CHB) is a global health issue which affects 350 million people.^[1,2]^ Although effective hepatitis B virus (HBV) vaccines have been around more than 30 years, the global morbidity of chronic HBV infection is decreased slightly, from 4.2% in 1990 to 3.7% in 2005. Nevertheless, the absolute amount of chronic HBV infections increased from 223 million in 1990 to 240 million in 2005 worldwide.^[3]^ CHB is the primary cause of chronic hepatitis, cirrhosis, and hepatocellular carcinoma (HCC) globally.^[4]^ 25% to 30% of people infected with HBV may develop progressive liver fibrosis, which eventually leads to cirrhosis (end-stage liver disease), increasing the risk of HCC, and even death.^[5,6]^ In addition to morbidity and mortality, patients infected with HBV also have various physical, psychological, and social problems that reduce their quality of life.^[3,7]^ HBV infection also poses a high economic burden because most CHB patients are of working age.^[8,9]^

At present, most of the HBV drugs in clinical treatment have inhibitory effects on the life cycle of the virus. The currently HBV drugs included 7 medications: 2 formulations of interferon (IFN), standard and pegylated, and 5 nucleos(t)ide analogues: lamivudine, telbivudine, entecavir, adefovir, and tenofovir.^[10,11]^ These drugs can inhibit the replication of hepatitis B virus, improve liver inflammation, but not eradicate hepatitis B virus. Although the course of IFN is limited, the course of nucleos(t)ide analogues is multiyear and even requires lifelong medication. Long durations of treatment will increase the cost, the risk of adverse reactions and drug resistance, and is prone to relapse after withdrawal.

Although the name of CHB is not recorded in ancient Traditional Chinese Medicine (TCM) literature, similar records can be found according to the characteristics and clinical manifestations of the disease, which falls into the category of diseases such as jaundice, hypochondriac pain, and consumptive disease in TCM. For a long time, the therapeutic effect and beneficial contribution of TCM to CHB have been gradually recognized and confirmed in clinical practice and basic research progress.

Yinchenhao decoction (YCHD), originated from S*hanghan Zabing Lu*n, is composed of Yinchenhao (*Artemisiae Scopariae* Herba), Zhizi (*Gardeniae Fructus*), and Dahuang (*Radix Rhei et Rhizoma*). YCHD has a broad application prospect in the field of liver diseases. Experiments have shown that YCHD can promote bilirubin metabolism, prevent liver injury, inhibit liver cell apoptosis, activate hepatic stellate cell (HSC), and synthesize collagen in jaundice hepatitis^[12]^; YCHD can inhibit hepatic steatosis, reduce hepatic fat deposition, and protect liver function in nonalcoholic steatohepatitis (NASH)^[13]^; YCHD can alleviate liver fibrosis in an experimental liver fibrosis rat model.^[14–16]^ Clinical studies have shown that YCHD is worthy of clinical promotion and plays crucial roles in CHB treatment, significantly improving the clinical symptoms and viral replication of patients, and restoring liver function.^[17–19]^

Although many clinical trials have reported that the treatment of CHB by YCHD can enhance clinical effects and decrease the relapse rate of complications, the sample size of each study is relatively small, and the reported differences are large and weak. As a result, YCHD is not considered a reliable therapy for CHB internationally. On this basis, the purpose of this systematic review and meta-analysis is to evaluate the clinical effects of YCHD in the treatment of CHB and to provide clinicians with a medical basis for inquiry.

## 2 Methods

### 2.1 Inclusion criteria for study selection

#### 2.1.1 Types of studies

We will include all randomized controlled trials (RCTS) related to YCHD treatment of CHB. No language or release status constraints will be set.

#### 2.1.2 Types of participants

Participants who are clinically diagnosed with CHB, according to the APASL Guideline of Prevention and Treatment for Chronic Hepatitis B (2015 Update),^[20]^ will be included. Participants in this study will not have gender, ethnicity, economic status, age or educational restrictions.

#### 2.1.3 Types of interventions

The experimental group used a prescription containing YCHD as the main component and was orally administered. The dosage form can be a decoction, granule, or other modern dosage forms. Dosage and treatment are not limited. The control group can be a blank control, a placebo, or a conventional medication. If combined with traditional Chinese and Western medicine treatment, the Western medicine treatment of the control group should be consistent with the experimental group.

#### 2.1.4 Types of outcome measures

##### 2.1.4.1 Major outcomes

- Quantitative detection of HBV-DNA;
- Qualitative detection of HBeAg;
- The level of ALT.

##### 2.1.4.2 Secondary outcomes

- Syndrome according to standards for assessing TCM;
- Changes in participants’ life quality;
- Adverse events;
- Cost.

### 2.2 Search methods for the identification of studies

#### 2.2.1 Electronic searches

The purpose of this study is to search relevant papers on YCHD therapy for CHB in the electrical databases, including 4 Chinese databases (e.g., Wanfang database, Chinese National Knowledge Infrastructure (CNKI), Chinese Biomedical database (CBM), and Chinese Science and Technology Periodical database (VIP)) and 3 English databases (e.g., PubMed, Cochrane Library, and EMBASE). The literatures involved are those delivered from the time when the databases were established to January 2019. According to the Cochrane Handbook, a retrieval scheme for retrieving the PubMed database is given in File 1 and similar schemes will be adopted for other databases.

#### 2.2.2 Other resources search

A systematic review or meta-analysis of the relevant system for RCTs will be performed by electronic retrieval. In addition, relevant meeting minutes, eligible research reference lists, and gray literature will be manually searched for additional resources.

### 2.3 Data acquisition and analysis

#### 2.3.1 Selection

Two investigators will look through the headings and abstracts of the essays which were obtained based on previously incorporated exclusion criteria for unrelated literature; in addition, for articles that meet the accepting criteria, the reviewer will read through the entire paper to determine whether it meets the criteria. If there is any disagreement, it will be resolved by consulting other researchers. The missing information will be supplemented by connecting the original author.

#### 2.3.2 Data collection and management

Two investigators extracted information (e.g., disease duration, diagnosis, disease comorbidity, severity, sample size, age, gender, treatment plan, follow-up, outcome measures, study results, adverse events) from the literature that satisfy the inclusion criteria. When there is any doubt about the data, it should be resolved through group discussion, contacting author, or third-party arbitration.

#### 2.3.3 Assessment of risk of bias in included studies

One of the tools described in the Cochrane Intervention System Review Manual version 5.1 will be used to evaluate risk biases. The tool includes stochastic sequence creation, allocation concealment, blindness of participants and investigators, blindness of outcome assessment, incomplete result data, and selectivity of reporting results. Two investigators will independently assess the included trials’ methodological quality. There are 3 evaluation results (low risk, unclear, high risk). When there is any doubt about the data, it should be resolved through group discussion, contacting author, or third-party arbitration.

#### 2.3.4 Measures of treatment effect

For continuous data, the extracted data will be assessed using a standard mean difference (SMD) of 95% confidence interval (95% CI). For dichotomous outcomes, we will present a 95% CI ratio (RR) for analysis.

#### 2.3.5 Missing data

If there is no necessary information in the included literature, we will obtain complete information via email to the author of the main study. If no additional messages are received, we will use the available data for data synthesis. At the same time, we will also discuss the possible consequences of missing data in the review.

#### 2.3.6 Assessment of heterogeneity

In this study, *χ*^2^ test will be used to analyze the heterogeneity between the results of the study and heterogeneity was determined by quantitative binding to I^2^.^[21]^ If I^2^ >50%, there is a large heterogeneity between studies. If I^2^< = 50%, the heterogeneity between statistical studies is negligible. The effect size is estimated using a fixed effect model.

#### 2.3.7 Assessment of reporting bias

If the involved trial data is sufficient in the review (more than 10 pieces), we will place a funnel plot based on the Egger method, which is designed to discuss reported bias or small research effects.

#### 2.3.8 Publication bias

If the included study is sufficient in the review (more than 10 pieces), we will place a funnel plot based on the Egger method, which is designed to discuss publication bias.

#### 2.3.9 Data synthesis

The Review Manager (RevMan) software (V5.3, Cochrane Collaboration, Oxford, England) will be used to compute the data synthesis. In the absence of significant statistical heterogeneity, a fixed effect model will be used for analysis. In the presence of statistical heterogeneity, the observers will examine the source of statistical heterogeneity in further analysis. After excluding significant clinical heterogeneity, we will use a random-effect model for meta-analysis. If there is significant clinical heterogeneity, we will use subgroup analysis or sensitivity analysis, or just descriptive analysis.

#### 2.3.10 Subgroup analysis

If the included studies are sufficient (at least 10 trials), the observers will perform a subgroup analysis based on inconsistent participant characteristics, different control interventions, and outcome measures. The purpose of the subgroup analysis is to explore heterogeneous resources.

#### 2.3.11 Sensitivity analysis

The quality of the included literature will be examined by sensitivity analysis based on sample size, missing data results, and methodological quality.

#### 2.3.12 Grading the quality of evidence

The quality of evidence will be evaluated by the Grading of Recommendations Assessment, Development and Evaluation (GRADE) and rate it into 4 levels (high, moderate, low, or very low).^[22]^

#### 2.3.13 Ethics

The ethical approval is not necessary, because we will not use data related to individual patient data.

## 3 Discussion

**Figure 1:**
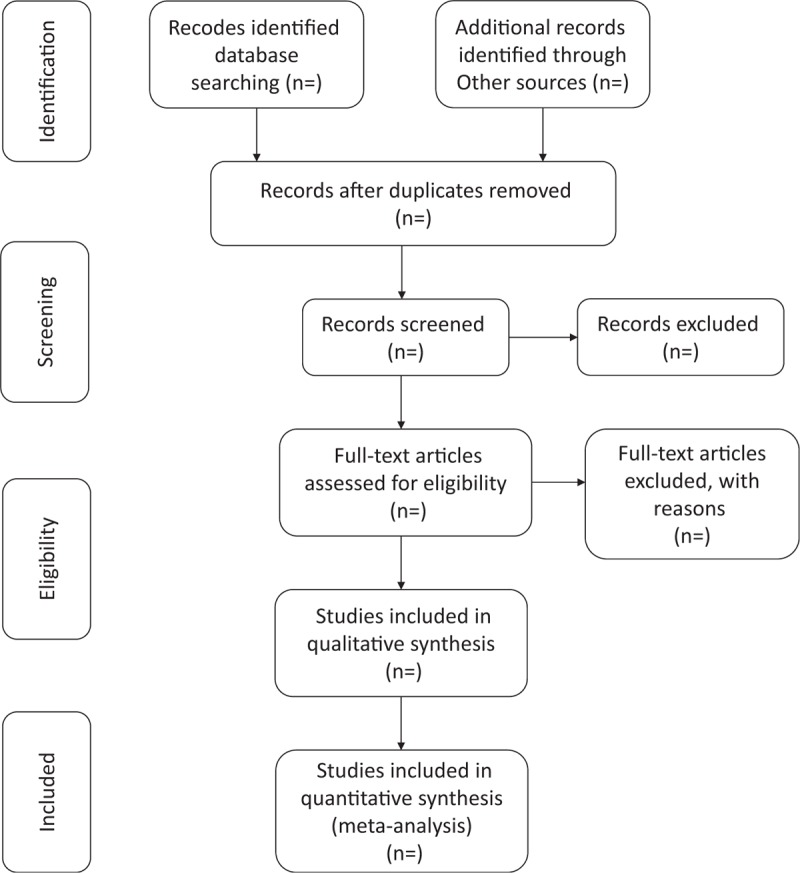
Flow diagram of study selection process.

CHB is a global public health problem with a huge social and economic burden. At present, the treatment of CHB is mainly antiviral, with long treatment time, large side effects, and recurrence after withdrawal. TCM has been used to treat CHB for more than 2000 years. YCHD is a classical Chinese medicine prescription for the treatment of jaundice. Although there have been some reports on the treatment of CHB with YCHD, the sample size is small. Its safety and effectiveness need to be further verified. Therefore, we have updated a systematic review to determine the efficacy and safety of YCHD for CHB with high-quality, large-sample evidence. The evaluation of this systematic review can be divided into 4 parts (identification, inclusion study, data extraction, and data synthesis, Figure 1). The purpose of this review is to provide more convincing evidence for clinicians’ decisions in the treatment of CHB patients. There are also potential shortcomings in this study. Different doses of YCHD included in CHB trials and efficacy evaluation criteria may lead to significant clinical heterogeneity.

## Author contributions

TS is the guarantor of the article. The manuscript was drafted by LX and TX. LX and TS developed the search strategy. LX, TX, and SJ will independently screen the potential studies and extract data and will also assess the risk of bias and finish data synthesis. TS will arbitrate any disagreement and ensure that no errors occur during the review. All review authors critically reviewed, revised, and approved the subsequent and final version of the protocol.

**Conceptualization:** Tao Shen.

**Data curation:** Lu Xu, Tian Xie, Shengnan Jian.

**Project administration:** Tao Shen.

**Software:** Lu Xu, Tian Xie.

**Writing – original draft:** Lu Xu, Tian Xie.

**Writing – review & editing:** Tao Shen.

## Supplementary Material

Supplemental Digital Content
