# Supplementary material for: Yinchenhao decoction for chronic hepatitis B: Protocol for a systematic review and meta-analysis
Source: Medicine (Baltimore). 2019 Feb 22;98(8):e14648. doi: 10.1097/MD.0000000000014648 (PMC6408079; doi:10.1097/MD.0000000000014648)
Supplement: Supplemental Digital Content [file medi-98-e14648-s001.doc]

**Appendix A.**

***Search strategy used in PubMed database***

#1 Yin-Chen-Hao-Tang OR Yinchenhao decoction

### #2 Chronic Hepatitis B OR Chronic Hepatitis B Virus Patients OR Chronic Hepatitis B Virus OR Chronic Hepatitis Infection OR [Chronic Hepatitis B Virus Infection OR Chronic B-related Hepatitis OR Hepatitis B Virus Chronic Carrier.](https://www.ncbi.nlm.nih.gov/pubmed/25400473)

#3 Randomized controlled trial OR clinical study OR Clin-ical Trial OR Controlled study OR Controlled Trial OR Random*Control* study OR random* Control* Trial

#1 AND #2 AND #3
